# Supplementary material for: Applied Methods to Assess the Antimicrobial Activity of Metallic-Based Nanoparticles
Source: Bioengineering (Basel). 2023 Oct 28;10(11):1259. doi: 10.3390/bioengineering10111259 (PMC10669044; doi:10.3390/bioengineering10111259)

Supporting Information

Applied methods to assess the antimicrobial activity of metallic based nanoparticles

*Etelka Chung, Guogang Ren, Ian Johnston, Rupy Kauru Mathar, Lena Ciric, Agnieszka Walecka and Yuen-Ki Cheong**

Figure S1: Example of agar well diffusion zone of inhibition. *S. aureus* bacteria with nanoparticle testing samples at 0.1wv%: a) CuZn, b) AgCu, c) antimicrobial metallic nanoparticle control, and d) antibiotic control (10 µg/ml gentamicin). To measure the zone of inhibition, the diameter was measured (as shown by the double ended arrow in b) in cm and the mean was calculated for the measurements taken from the triplicates.

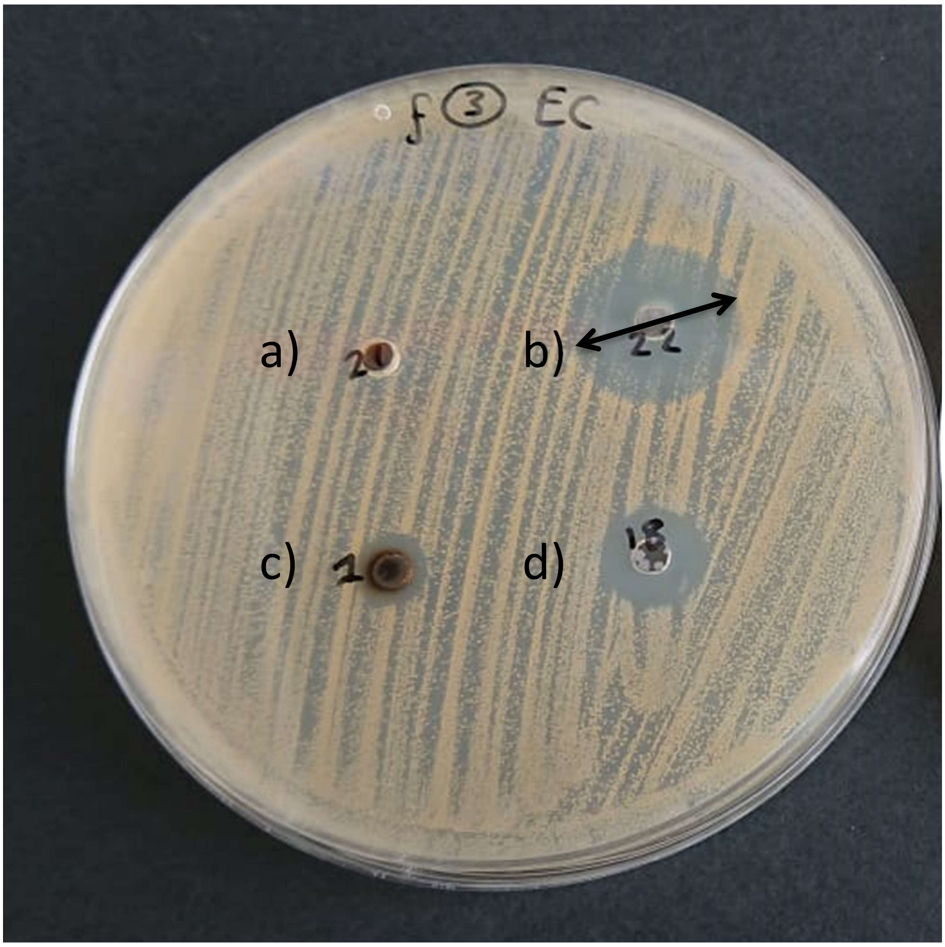

Figure S2: Sepctrometry absorbance of AgCu nanoparticles at concentrations between 500 μg/ml to 3.9 μg/ml to monitor optical density disturbance. Error bars represent the standard deviation.

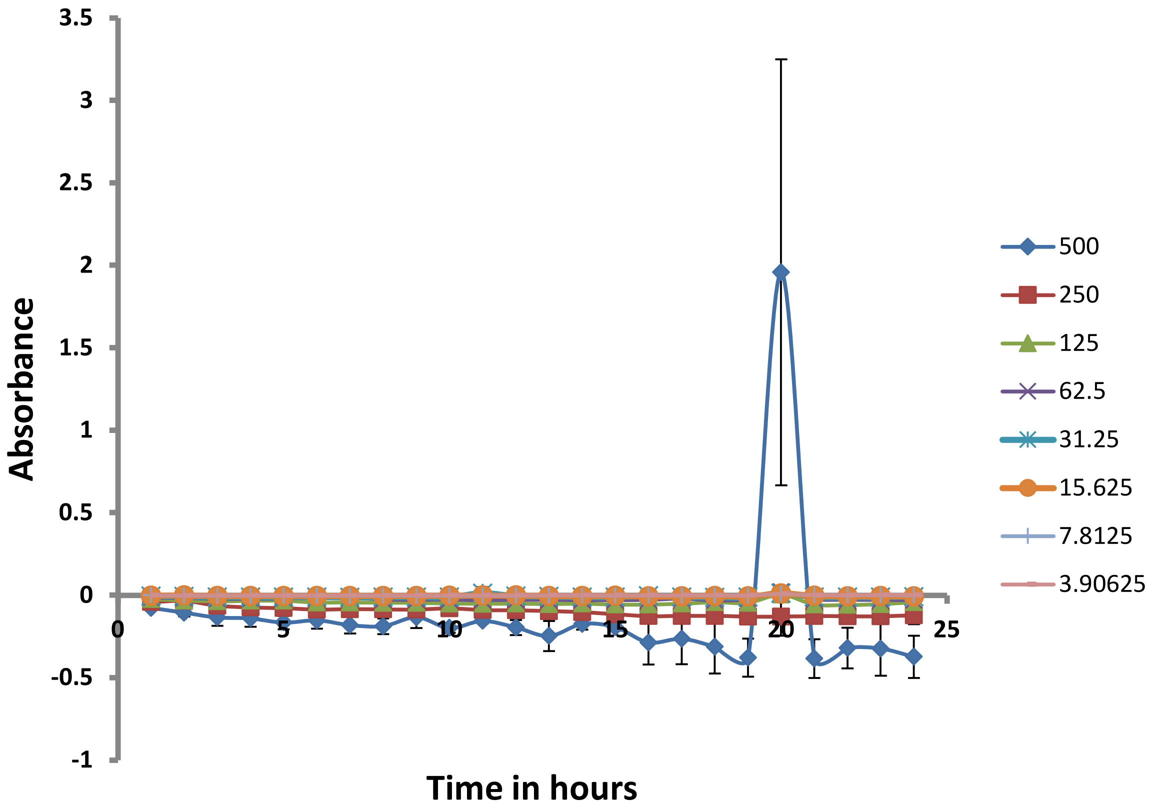

Figure S3: Well plate of MIC resutls. Each well plate contains 3 nanoparticle samples (4 columns for each sample) with 2 fold decreasing concentration gradient of nanoparticles as indicated on the right. Highlighted in white, is the first column of each sample, which is a nanoparticle control. The remainig 3 columns are triplicates of the sample. Highlighted in red is the MIC. Highlighted in black and blue is the positive and negative control, respectively. Lastly, in green is the antibiotic control, with decreasing concentration from left to right. Pink well colour indicates live viable cells that reduced resazruin to resorufin, whilst blue well colour indicates non viable cells.

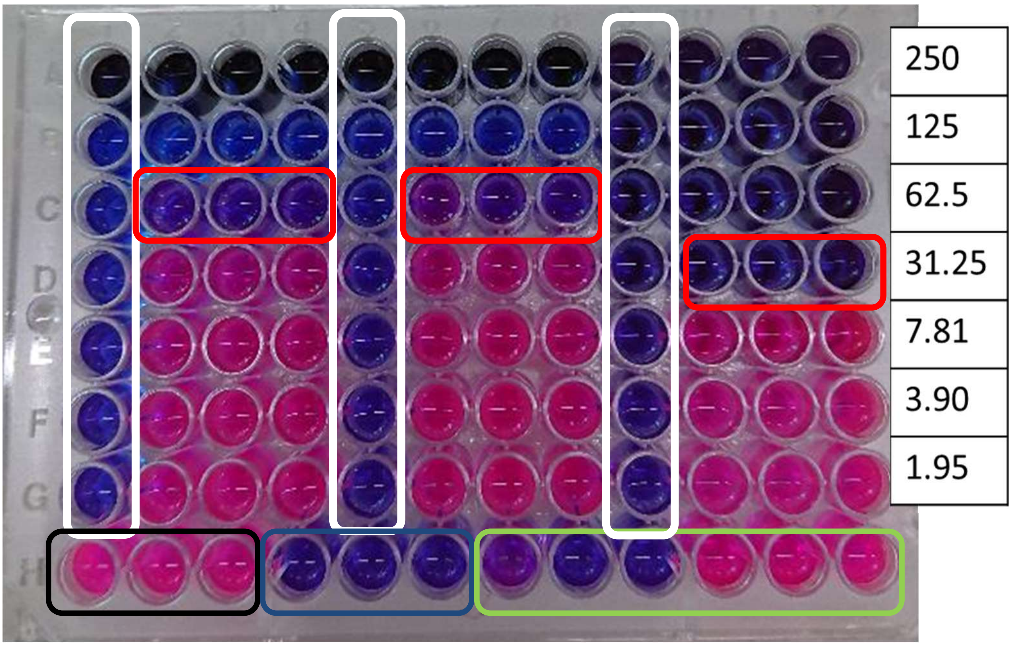

Figure S4: 24 hour kinetic growth curve of microbes after AgCu nanoparticle treatment at concentrations between 62.5 µg/ml to 0.5 µg/ml: a) *C. albicans,* b) *E. coli* and c) *S. aureus.* Error bars are represented using standard deviation.

a)

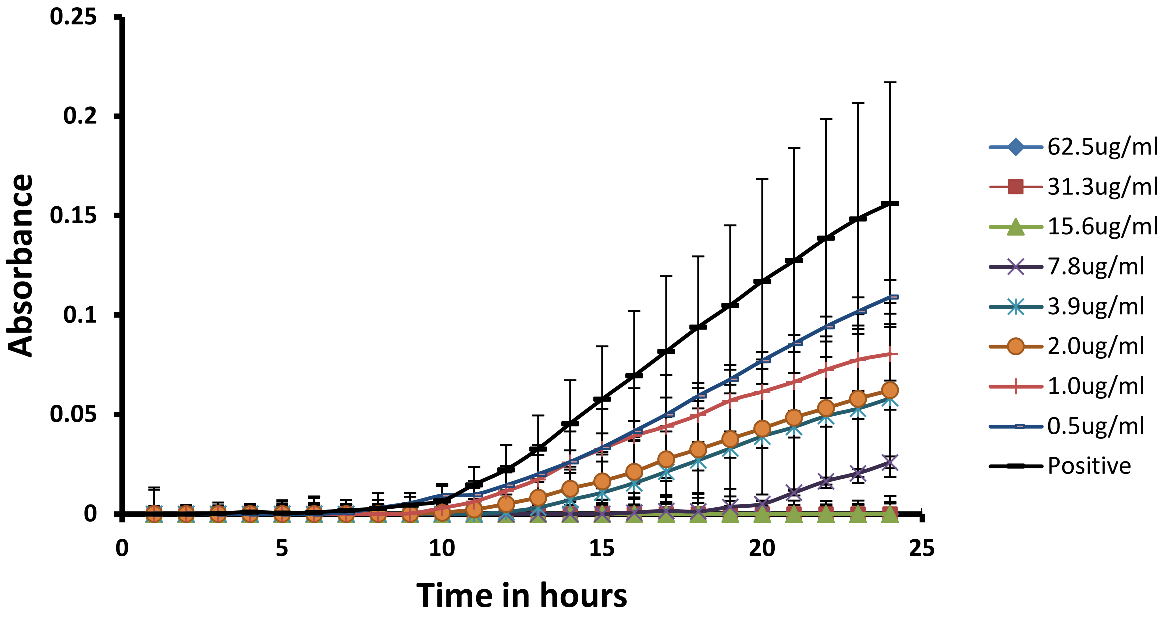

b)

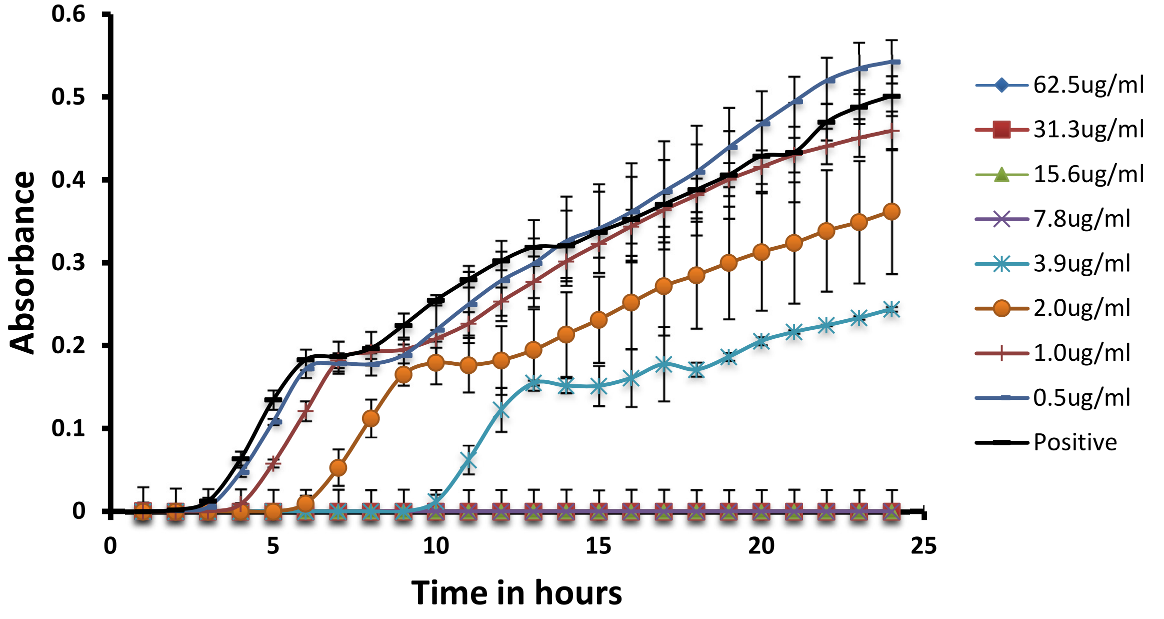

c)

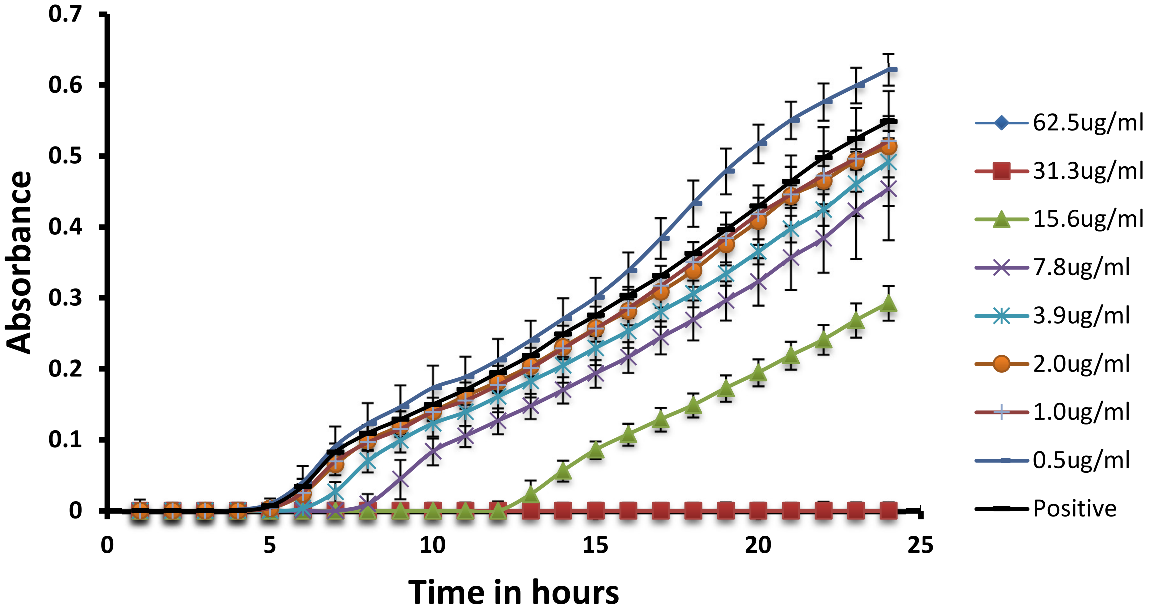

0.7
0.6
0.5
0.4
0.3
0.2
0.1
0
Absorbance
0
5
10
15
20
25
Time in hours
62.5ug/ml
31.3ug/ml
15.6ug/ml
7.8ug/ml
3.9ug/ml
2.0ug/ml
1.0ug/ml
0.5ug/ml
Positive

Figure S5: Percentage cell viability of microbes after exposures of different concentrations of AgCu nanoparticles (100 µg/ml and the corresponding MIC) over period of five hours. Results represent three areas of slide count of two replicates with error bars denoting half of the standard deviation.

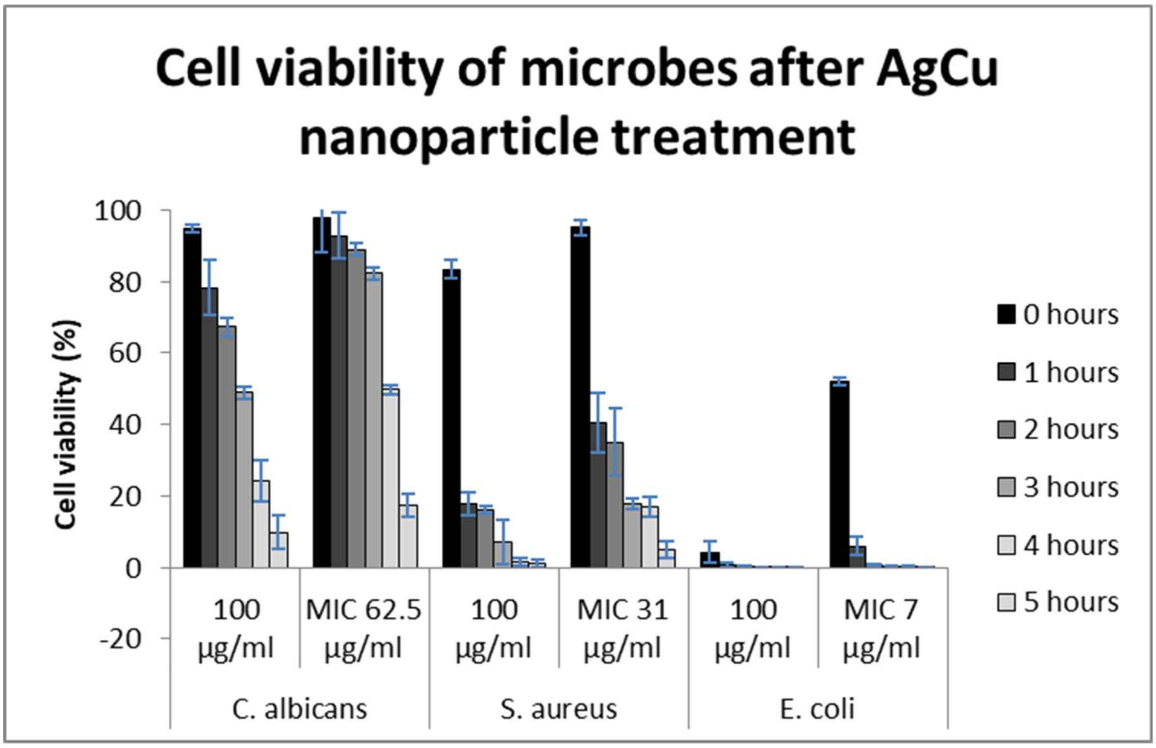

Supplement: Supplementary file 1 [file bioengineering-10-01259-s001.zip › bioengineering-2687325-supplementary.pdf]
